# A study on disability glare vision in young adult subjects

**DOI:** 10.1038/s41598-023-30658-0

**Published:** 2023-03-02

**Authors:** Pilar Casado, Francisco J. Ávila, Mª Victoria Collados, Jorge Ares

**Affiliations:** Departamento de Física Aplicada, Universidad de Zaragoza, 50009 Zaragoza, Spain

**Keywords:** Applied optics, Health care, Eye diseases

## Abstract

The full assessment of the visual system must include the evaluation of the optical quality of the eye and neural visual functions. The objective evaluation of the retinal image quality is often carried out by computing the point spread function (PSF) of the eye. The central part of the PSF is associated with optical aberrations and the peripheral areas with scattering contributions. In that sense, visual acuity and contrast sensitivity function tests can be considered the perceptual neural response to those contributions characterizing the eye’s PSF. However, in natural viewing conditions, visual acuity tests may provide good vision while contrast sensitivity tests can reveal visual impairment in glare vision conditions, such as exposure to bright light sources or night driving conditions. Here we present an optical instrument for the study of disability glare vision under extended Maxwellian illumination to assess the contrast sensitivity function under glare conditions. The limit of the Total Disability Glare threshold, tolerance, and glare adaptation will be investigated as a function of the angular size of the glare source (GA) and the contrast sensitivity function in young adult subjects.

## Introduction

Contrast Sensitivity (CS) is the ability of the visual system to perceive the relative luminance between an object and the background^1^. In healthy subjects, contrast sensitivity can be affected by aging, refractive error, or pupil diameter^2^ and its measurement depends on the ambient luminance level. Jindra and collaborators reported higher contrast sensitivity measurement in photopic conditions compared to scotopic light levels^1^, however, a more recent study concluded that scotopic conditions provide higher contrast sensitivity measures than photopic conditions^2^. Those discrepancies in CS measurements could be explained by diffraction and spherical aberration effects in photopic and scotopic conditions, respectively^3^.

Furthermore, the presence of bright light sources in the visual field may cause light-scattering within the ocular media^4^ known as ocular straylight. In normal eyes, ocular straylight mainly depends on the ocular media (cornea, sclera, iris, crystalline lens, and retinal fundus), age, and the illuminance of the light source^5^. Moreover, pathological conditions such as corneal edema and dystrophy^6^ or cataracts^7^ can increase ocular straylight drastically limiting the patient’s visual quality and quality of life. Ocular straylight can be measured by optical^8^ and psychophysical approaches^9^. The main optical effect of ocular straylight is the generation of a light veil distribution (veiling glare)^10^ in the retina capable of inducing a total loss of retinal image contrast^11^ which is known as Total Disability Glare^12^. However, bright light sources are also related to another type of glare which is not always accompanied by visual impairment, the Discomfort Glare^13^.

The research into the relationship between visual comfort and visual environment has resulted in the proposal of some luminance-based metrics^14,15^. These metrics have been quite successful in predicting Glare Discomfort. CIE Glare index (CGI), Visual Comfort Probability (VCP), and Unified Glare Rating (UGR) are efficient tools helping architects and engineers to design natural and artificial lighting related to buildings^16^, streets^17^, and roads^18^.

However, despite the things they share, Discomfort Glare and Total Disability Glare must be understood as two different phenomena. In fact, the empirical evidence indicates that Discomfort Glare indexes are mainly dependent on the ratio between the luminance of the objects present in the visual field and the luminance of the background, since Total Disability Glare depends on the total amount of light that enters the eye (measured as the illuminance at the cornea plane) causing straylight. It is in this regard that straylight can be understood as a measure of ocular media clarity^19^ while Disability Glare accounts for the intraocular scattering effects on visual performance. According to the *Commission Internationale de l’Eclairage* (CIE), there are four psychophysical procedures for disability glare assessment: category rating, discrimination, adjustment, and matching^9^. All the tests devoted to evaluating Total Disability Glare share the same simple principle: assessment of the visual performance (typically visual acuity and CS) with and without the presence of an external glare source.

In this work, we present a psychophysical version of an objective wide-field ocular straylight meter^8^ for the assessment of the disability glare vision. Moreover, taking advantage of this instrument, a study on Total Disability Glare as a function of sinusoidal gratings of different spatial frequencies, corneal illuminance and angular field size of a circular glare source is also presented.

## Results

### Total disability glare illuminance

**Figure 1:**
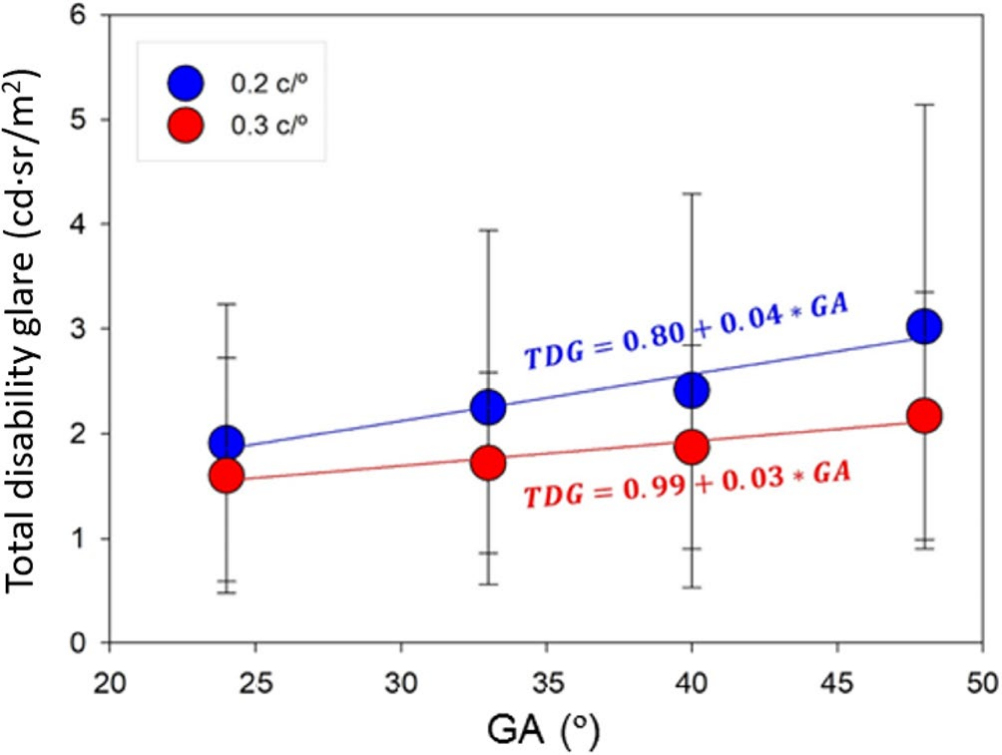
Graphical representation of the TDG parameter as a function of angular size of the glare source (GA) and linear regression for spatial stimulus frequencies of 0.2 ($R^{2} = 0.943, \,p = 0.029$) and 0.3 c/° ($R^{2} = 0.942,\, p = 0.029$).

Table 1 shows the average and standard deviation for the Total Disability Glare (TDG) illuminance for all subjects involved in the study, obtained for the different spatial frequencies of the stimulus and angular fields of the glare source. The maximum TDG value found (3.02 ± 1.88 cd·sr/m^2^) corresponded to the minimum spatial frequency (0.2 c/°) evaluated and the maximum angular size of the glare source (48°), while the minimum TDG value (1.26 ± 0.92 cd sr/m^2^) was obtained for the maximum spatial frequency (1.8 c/°) and the minimum angular size of the glare source (24°). The analysis of variance and the subsequent Tukey’s test showed significant differences between the lowest frequency and the rest of the frequencies. However, no significant differences were found between the results for frequencies higher than 0.3 c/°, so we represent in Fig. 1 the mean TDG illuminance as a function of the angular field of the glare source only for the spatial frequencies of 0.2 and 0.3 c/°.

**Table 1:** Mean ± standard deviation in [cd m^2^] of the TDG parameter for all spatial frequencies and angular sizes of the glare source.

GA	Total Disability Glare parameter [cd sr/m^2^]					
	0.2 c/°	0.3 c/°	0.6 c/°	0.9 c/°	1.2 c/°	1.8 c/°
24°	1.91 ± 1.32	1.60 ± 1.12	1.29 ± 0.77	1.37 ± 0.79	1.44 ± 0.96	1.26 ± 0.92
33°	2.25 ± 1.69	1.72 ± 0.86	1.78 ± 1.22	2.19 ± 1.32	1.73 ± 0.91	1.67 ± 1.03
40°	2.41 ± 1.88	1.87 ± 0.97	1.57 ± 1.05	1.90 ± 1.22	2.02 ± 1.30	1.79 ± 1.16
48°	3.02 ± 1.88	2.17 ± 1.18	1.68 ± 1.05	1.88 ± 1.03	1.94 ± 1.12	1.68 ± 1.06

Results in Fig. 1 show that the higher the angular distribution of the glare discs, the higher the TDG illuminance. The linear fits for 0.2 and 0.3 c/° frequencies were TDG = 0.80 + 0.04*GA ($$R^{2} = 0.943, p = 0.029$$) for 0.2c/° and TDG = 0.99 + 0.03*GA ($$R^{2} = 0.942, p = 0.029)$$, respectively. Although the data obtained for frequencies higher than 0.3 c/° are not represented, we obtained that the linear correlation between GA and TDG disappears in those cases.

### Glare tolerance

**Figure 2:**
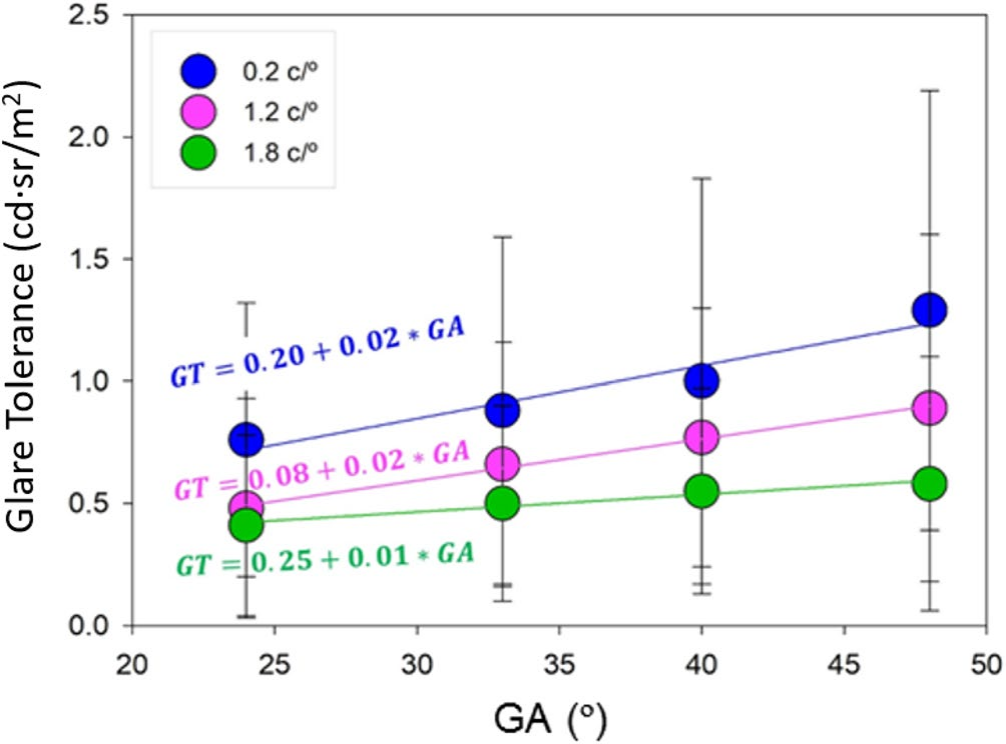
Graphical representation of the GT parameter as a function of angular size of the glare source and linear regression for spatial stimulus frequencies of 0.2 (blue), 1.2 (purple) and 1.8 c/° (green).

The average values and standard deviation obtained for the glare tolerance (GT) are presented in Table 2. As for the TDG parameter, the maximum value of the GT parameter (1.29 ± 0.90 cd sr/m^2^) is found for the minimum spatial frequency and the maximum angular field, whereas the minimum value (0.41 ± 0.37 cd sr/m^2^) is obtained for the maximum frequency and the minimum angular field. The analysis of variance showed significant differences between the frequencies analyzed but, unlike the TDG parameter, Tukey's test revealed that there are not statistical significant differences between the frequencies 0.2 c/° and 0.3 c/° and between the frequencies 0.6 c/°, 0.9 c/°and 1.8 c/°. The frequency 1.2 c/° presents significant differences with all of the other frequencies, so we represent in Fig. 2 the mean values of the GT parameter as a function of the angular field of the glare source for frequencies 0.2 c/°, 1.8 c/°, and 1.2 c/°.

**Table 2:** Mean ± standard deviation in [cd sr/m^2^] of the GT parameter for all spatial frequencies and angular sizes of the glare source.

GA	Glare tolerance parameter [cd sr/m^2^]					
	0.2 c/°	0.3 c/°	0.6 c/°	0.9 c/°	1.2 c/°	1.8 c/°
24°	0.76 ± 0.56	0.79 ± 0.52	0.52 ± 0.38	0.53 ± 0.42	0.48 ± 0.45	0.41 ± 0.37
33°	0.88 ± 0.71	0.84 ± 0.59	0.64 ± 0.42	0.76 ± 0.50	0.66 ± 0.50	0.50 ± 0.40
40°	1.00 ± 0.83	0.83 ± 0.54	0.73 ± 0.62	0.72 ± 0.45	0.77 ± 0.53	0.55 ± 0.42
48°	1.29 ± 0.90	1.02 ± 0.76	0.70 ± 0.55	0.72 ± 0.47	0.89 ± 0.71	0.58 ± 0.52

Positive linear correlation of the GT parameter with the angular size of the glare source was observed for the three spatial frequencies: GT = 0.20 + 0.02*GA ($R^{2} = 0.937, \,p = 0.032$) for 0.2 c/°, GT = 0.08 + 0.02*GA ($R^{2} = 0.995, \,p = 0.003$) for 1.2 c/°, and GT = 0.25 + 0.01*GA ($R^{2} = 0.958,\, p = 0.021$) for 1.8 c/°.

### Disability acceptance parameter

**Figure 3:**
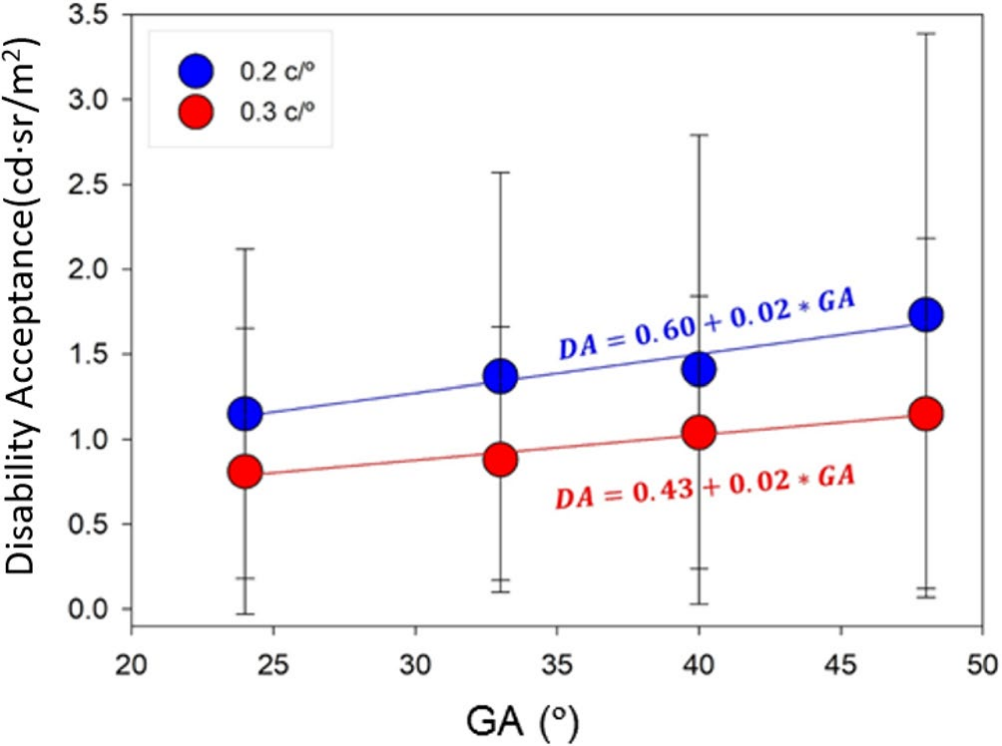
Graphical representation of the DA parameter as a function of angular size of the glare source and linear regression for spatial stimulus frequencies of 0.2 (blue) and 0.3 c/° (red).

The disability acceptance parameter was defined as that range of illuminance at the pupil plane that, without causing Total Disability Glare, produces a decrease in contrast sensitivity but still allows the perception of some spatial frequencies. Table 3 shows the mean and standard deviation of this parameter, obtained from the difference between TDG and glare tolerance. Analysis of variance and Tukey's test revealed significant differences between the frequency of 0.2 c/° and the rest of the frequencies. There were found, however, no significant differences between the spatial frequencies higher than 0.3 c/°. However, no significant differences were found between the results for frequencies higher than 0.3 c/°, so we represent in Fig. 3 the mean DA illuminance as a function of the angular field of the glare source only for the spatial frequencies of 0.2 and 0.3 c/°. The linear regression for these frequencies was: DA = 0.60 + 0.02*GA ($R^{2} = 0.933,\, p = 0.034$) and DA = 0.43 + 0.02*GA ($R^{2} = 0.965,\, p = 0.018$).

**Table 3:** Mean ± standard deviation in [cd sr/m^2^] of the DA parameter for all spatial frequencies and angular sizes of the glare source.

GA	Disability acceptance parameter [cd sr/m^2^]					
	0.2 c/°	0.3 c/°	0.6 c/°	0.9 c/°	1.2 c/°	1.8 c/°
24°	1.15 ± 0.97	0.81 ± 0.84	0.77 ± 0.68	0.85 ± 0.56	0.96 ± 0.72	0.85 ± 0.77
33°	1.37 ± 1.20	0.88 ± 0.78	1.14 ± 1.01	1.43 ± 1.16	1.07 ± 0.84	1.17 ± 0.76
40°	1.41 ± 1.38	1.04 ± 0.80	0.85 ± 0.71	1.18 ± 1.14	1.25 ± 0.95	1.24 ± 0.89
48°	1.73 ± 1.66	1.15 ± 1.03	0.98 ± 0.76	1.16 ± 0.77	1.05 ± 0.75	1.11 ± 0.81

## Discussion

The optical quality of the eye has been traditionally assessed by analyzing its point spread function (PSF) as it contains information on both optical aberrations and scattering effects^20^. While aberrations are associated with the central part of the PSF, the periphery is associated with scattering contributions that, according to the general glare equation that describes disability glare, has a validity angular domain from 0.1° to 100° in the human eye.

However, the visual function assessment must also include not only an evaluation of the retinal image quality (objective optical assessment) but considers the neural contrast sensitivity^21^. While visual acuity tests may confirm good vision quality under natural viewing conditions, the contrast sensitivity function may only manifest visual impairment under disability glare conditions or when larger retinal areas are exposed by glare sources, such as night driving^22,23^.

Here we present a modification of an objective wide-field ocular straylight meter^8^ to assess the visual function under disability glare conditions. The system operates in extended Maxwellian-illumination projecting the center of the glare source and the visual stimuli directly onto the fovea^24^.

The optical system controls the angular distribution and the illuminance of the glare source, whereas the visual stimuli, consisting of sinusoidal gratings with variable spatial frequency and contrast, are computer generated.

Disability glare concept was explored by measuring the contrast sensitivity function under glare vision conditions in young healthy subjects. First, the CS function was evaluated for each subject across a range of spatial frequencies from 0.2 to 1.8 c/° to establish a glare-free baseline vision. Next, the subject was exposed to a glare source of increasing illuminance to study the Total Disability Glare threshold (the point at which the glare source causes temporary blindness), the recovery of the baseline vision, and the amount of glare the subject can tolerate while maintaining the contrast sensitivity function.

Results revealed that Total Disability Glare illuminance is correlated to the incident angular size of the glare source, that is, the higher the angular distribution of the glare discs the higher the corneal illuminance threshold required to get Total Disability Glare is. Our results are in agreement with those reported by Vos^25^, they found that the variation of equivalent veiling luminance as a function of the corneal illuminance strongly decreases as the incident glare source angle increases. Our results also showed that this dependence is statistically significant for the smallest spatial frequencies only.

The glare tolerance analysis corresponding to the glare regime at which the baseline CS function is recovered, revealed that the tolerance to a glare source is positively correlated to the angular size of the disc of the glare source (See Fig. 1) but unlike the TDG illuminance threshold, significant correlations were found for almost all the analyzed spatial frequencies. Moreover, the higher the spatial frequency, the lower the variation of the glare tolerance as a function of the angular size of the glare source (See Fig. 2).

Finally, results showed that once the glare tolerance level is reached, the disability acceptance decreases as the angular size of the glare source approaches the central retina (Fig. 3). However, disability acceptance was found to be statistically dependent in accordance with TDG results shown in section 3.1) with the incident angle of the source for the smallest spatial frequencies of 0.2 and 0.3 c/°.

To summarize, the shift in Total Disability Glare illuminance threshold, glare tolerance and disability acceptance parameter as a function of the spatial frequency increases for larger angular distribution of the variable discs modified at the glare source. Then, disability glare vision depends not only on the illuminance of the source and the angular distribution of the incident glare source, but the spatial frequency of the observed stimuli.

Nevertheless, the study has some potential limitations which include the lack of control of individual pigmentation type among participants. As it is well-known, pigmentation type is an important factor to straylight^10^ so it is possible that some of our results can only be associated with the pigmentation type of our sample. Moreover, it can also be argued that the spatial frequency range used in this work (low spatial frequencies from 0.2 to 1.8 c/°) might not be adequate when a typical human eye can resolve spatial frequencies up to 30–40 c/°. However, it is important to remark that the low spatial frequencies are the most important region of the spectrum in order to localize the surrounding objects. Following this line, our results could be useful to predict how glare could affect the visual performance for object detection. Finally, it could have been interesting to measure stray light from our subjects with a standard method, with this information more insight could be obtained from our present results. However, it is considered that the present experiment gives by itself very interesting information about vision performance in glare conditions.

To conclude, a new psychophysical optical instrument has been developed for the study of disability glare regime and the threshold at which glare vision becomes in temporal blindness, as well as the amount of glare that is tolerated by the visual system in young adult subjects. The instrument allowed the definition of disability glare concepts involving factors such as angular distribution of the glare discs, the illuminance of the glare source, and the contrast sensitivity function. Future work includes an extension of the analyzed range of spatial frequencies to study the disability glare parameters herein reported as a function of age and different ocular pathological conditions such as macular affectations, intraocular opacifications, or unwanted glare consequences from refractive surgery.

## Methods

### Subjects

A total of 30 subjects were recruited in this study and measured by an experienced optometrist at the Laboratory of Visual Optics Research of the University of Zaragoza (Spain). The average age was 28.1 ± 9.3 years old, and the average equivalent spherical error was − 1.72 ± 2.18. Exclusion criteria included the presence of any ocular pathology and history of ocular surgery.

The study protocol adhered to the tenets of the Declaration of Helsinki and received the approval of the local Ethical Committee of Research of the Health Sciences Institute of Aragon, with reference C.P.-C.I.PI20/377. All subjects were informed about the experimental procedures of the research and signed an informed consent form prior to the start of the measurements.

### Disability glare instrument

**Figure 4:**
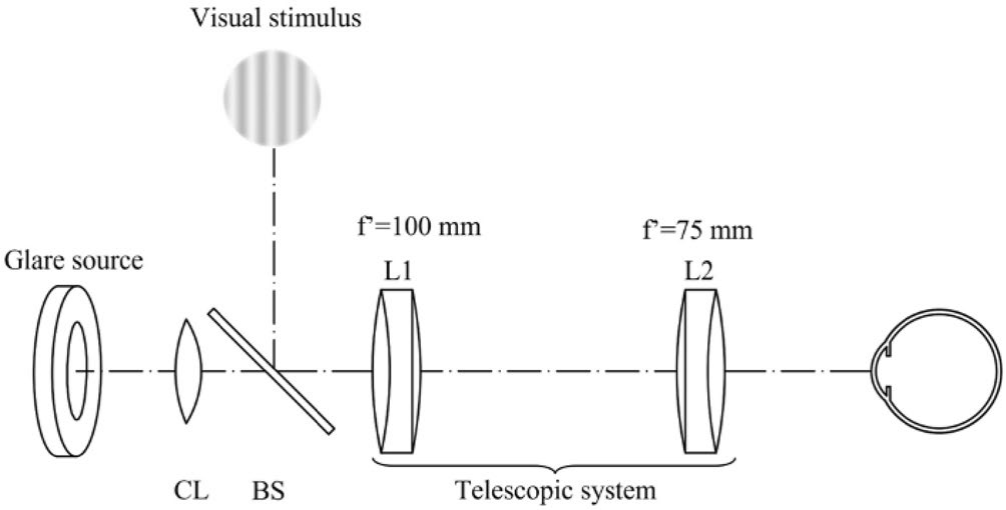
Optical layout of the experimental system: *CL* collimating lens, *BS* beam splitter.

A custom-made experimental system shown in Fig. 4 was developed with the aim of illuminating the retina with wide-angle distribution light in a coaxial way to the observation of a luminous sinusoidal stimulus. The system includes a circular glare source composed of a holographic light shaping diffuser (Light Shaping Diffusers, Luminit, LLC) back-illuminated by a white LED (Cree LED XLamp XM-L2). The luminous intensity of the source is controlled by an external dimmer. The angular distribution of the source is modified by means of removable 3D-printed masks with different angular apertures. This configuration creates uniform discs instead of the conventional annuli configurations^7^. Throughout the manuscript, we refer to angular size as the angular distribution of the uniform discs. For this experiment, four masks were used (with diameters 12.6 mm, 17.73 mm, 21.81 mm, and 26.67 mm), so the angular sizes subtended by the glare source once it was throughout the system were 24°, 33°, 40°, and 48°.

A relay telescopic system consisting of two achromatic doublet lenses conjugates the aperture of CL (45 mm focal length) with the pupil plane of the subject providing extended Maxwellian illumination. Then, the glare source and the CS test are directly projected on the retina minimizing unwanted factors affecting the disability glare measurement such as the pupil size and scattering contributions from iris and sclera^27^.

The visual stimuli consist of a contrast sensitivity test generated by Contrast-Test software (Opto-software). The software generates sinusoidal gratings that, through the system, present a total angular size of 9.8° with spatial frequencies of 0.2, 0.3, 0.6, 0.9, 1.2, and 1.8 cycles per degree (c/°) and contrast levels of 20, 11.1, 5.5, 3.12, 1.66, 0.87, 0.47, 0.25 and 0.12%. The luminance values of the test range from 46.5 cd/m^2^ for gratings with minimum contrast to 20.9 cd/m^2^ for grating with maximum contrast, so photopic conditions are maintained independently of the glare source luminance. A 50:50 beam splitter allows coaxial pathway of the visual target (displayed on an LCD monitor) and the glare source. The whole optical system was mounted on a 30 × 45 cm optical board coupled to a chin rest to facilitate alignment of the system axis with the subject's visual axis.

### Experimental procedure

The experiment was performed in scotopic conditions (to ensure the absence of external glare sources) and monocularly with all the subjects wearing their usual refractive correction. Before starting the measurements, 5 min were allowed for dark adaptation. The subject’s visual axis was kept aligned with the optical axis of the glare source and observation systems by means of the chin rest positioning controls.

The experiment involved first the estimation of a set of minimum perceptible contrast levels (MPCL) with the glare -source off for 6 different spatial frequency sinusoidal stimuli: 0.2, 0.3, 0.6, 0.9, 1.2, and 1.8 c/°. To accomplish that, the examiner started showing a sinusoidal stimulus with subthreshold contrast so the observers were not able to perceive any contrast on it. Then the contrast was progressively increased until the observer was able to perceive some contrast on it. The aim of this first step was to find a visual stimulus with a minimum but perceptible contrast. The mean values of minimum perceptible contrast obtained for each frequency are shown in Table 4.

**Table 4:** Mean ± standard deviation of the minimum perceptible contrast obtained for each spatial frequency.

Minimum perceptible contrast					
0.2 c/°	0.3 c/°	0.6 c/°	0.9 c/°	1.2 c/°	1.8 c/°
1.71 ± 0.43%	0.83 ± 0.12%	0.52 ± 0.12%	0.49 ± 0.11%	0.55 ± 0.31%	0.58 ± 0.33%

Secondly, while the observer was looking at this near-threshold sinusoidal pattern, the illuminance of the glare source was progressively increased to reach the minimum illuminance level able to avoid sinusoidal contrast recognition. The measurement of this illuminance level in the corneal observer plane was identified in this work as a *Total Disability Glare illuminance*.

**Figure 5:**
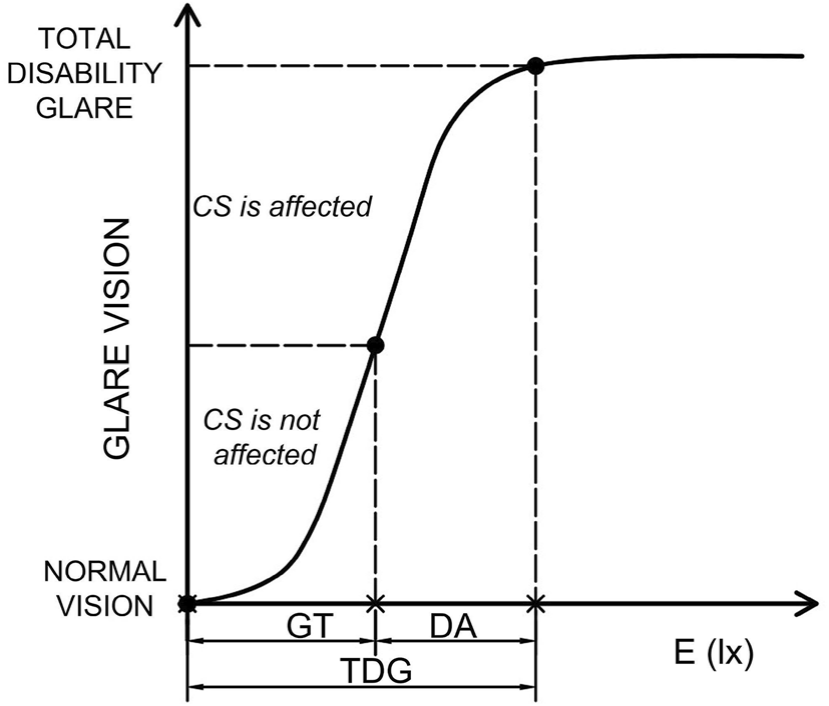
Schematic drawing of the Total Disability Glare illuminance (TDG), glare tolerance (GT), and disability acceptance (DA) parameter. The x-axis represents the illuminance at the pupil plane, and the y-axis represents the stages of glare vision.

After this first glare source illuminance level was determined, the examiner proceeded to slightly increase the illuminance level just before beginning a slow and progressive decrease of the illuminance level of the glare source to determine a second characteristic glare level just when sinusoidal contrast perception was recovered. This illuminance level will be named in this work as *glare tolerance*. From Total Disability Glare illuminance and glare tolerance, the *disability acceptance parameter* is defined in this work as the illuminance values that exceed the glare tolerance but are lower than the Total Disability Glare illuminance. This illuminance range was defined as the disability acceptance parameter and was obtained from the difference between the Total Disability Glare parameter and the glare tolerance. Figure 5 shows a drawing to facilitate the comprehension of the defined parameters.

This second step was repeated for each spatial frequency at 4 different glare angular distribution conditions (24, 33, 40, and 48 degrees). This meant 48 photometric level measurements for the glare source at the pupil plane for each participant (6 spatial frequencies × 4 glare angular sizes × 2 characteristic parameters).

### Data analysis

For each parameter defined above (TDG, GT y DA), one-way analysis of the variance and posterior Tukey test were carried out to check for significant differences in the values obtained as a function of the angular size of the glare source between each spatial frequency. Based on this statistical analysis, graphical representation of the mean parameters as a function of the angular field of the glare source is shown only for the spatial frequencies that present significant differences between them. Both graphical representations and statistical analysis were carried out using Sigmaplot (Systat Software, Inc, USA).

## Data Availability

Data herein reported are fully available in Tables 1, 2, 3, and 4.
